# Older Adults’ Attitudes Toward Deprescribing in 14 Countries

**DOI:** 10.1001/jamanetworkopen.2024.57498

**Published:** 2025-02-10

**Authors:** Renata Vidonscky Lüthold, Katharina Tabea Jungo, Kristie Rebecca Weir, Limor Adler, Radost Asenova, Sara Ares-Blanco, Markus Bleckwenn, Thomas Frese, Gilles Henrard, Aisling A. Jennings, Donata Kurpas, Vanja Lazic, Heidrun Lingner, Stina Mannheimer, Anabela Pereira, Ferdinando Petrazzuoli, Rosalinde K. E. Poortvliet, Ágnes Szélvári, Dorothea Wild, Emily Reeve, Zsofia Rozsnyai, Sven Streit

**Affiliations:** 1 Institute of Primary Health Care, University of Bern, Bern, Switzerland; 2 Graduate School for Health Sciences, University of Bern, Bern, Switzerland; 3 Division of Pharmacoepidemiology and Pharmacoeconomics and Center for Healthcare Delivery Sciences, Department of Medicine, Brigham and Women’s Hospital and Harvard Medical School, Boston, Massachusetts; 4 Sydney School of Public Health, Faculty of Medicine and Health, University of Sydney, Sydney, Australia; 5 Department of Family Medicine, Faculty of Medical & Health Sciences, Tel Aviv University, Tel Aviv, Israel; 6 Department of Urology and General Practice, Faculty of Medicine, Medical University of Plovdiv, Plovdiv, Bulgaria; 7 Federica Montseny Health Centre, Gerencia Asistencial Atención Primaria, Servicio Madrileño de Salud, Madrid, Spain; 8 Instituto de Investigación Sanitaria Gregorio Marañón, Madrid, Spain; 9 Institute of General Practice, Faculty of Medicine, Leipzig University, Leipzig, Germany; 10 Institute of General Practice and Family Medicine, Martin Luther-University Halle-Wittenberg, Halle (Saale), ST, Germany; 11 Department of General Practice, Faculty of Medicine, University of Liège, Liège, Belgium; 12 Department of General Practice, University College Cork, Cork, Ireland; 13 Division of Research Methodology, Department of Nursing, Faculty of Nursing and Midwifery, Wrocław Medical University, Wrocław, Poland; 14 Health Center Zagreb–Centar, Zagreb, Croatia; 15 Hannover Medical School, Center for Public Health and Healthcare, Department for Medical Psychology, Hannover, Germany; 16 Institute of Health and Care Sciences, Sahlgrenska Academy, University of Gothenburg, Gothenburg, Västra Götaland Region, Sweden; 17 Center for Health Technology and Services Research, Department of Education and Psychology, University of Aveiro, Campus Universitário de Santiago, Aveiro, Portugal; 18 Institute of Biomedical Sciences Abel Salazar, University of Porto, Porto, Portugal; 19 Sezione SNaMID Caserta, Caserta, Italy; 20 Center for Primary Health Care Research, Department of Clinical Sciences, Lund University, Malmö, Sweden; 21 Department of Public Health and Primary Care, Leiden University Medical Center, Leiden, the Netherlands; 22 LUMC Center for Medicine for Older People, Leiden University Medical Center, Leiden, the Netherlands; 23 Department of Family Medicine, Semmelweis University, Budapest, Hungary; 24 Institute of Family Medicine and General Practice, University Hospital Bonn, Bonn University, Bonn, Germany; 25 Centre for Medicine Use and Safety, Faculty of Pharmacy and Pharmaceutical Sciences, Monash University, Melbourne, Victoria, Australia; 26 Clinical and Health Sciences, University of South Australia, Adelaide, South Australia, Australia

## Abstract

**Importance:**

Better understanding of patients’ attitudes toward deprescribing specific medications will inform future deprescribing interventions.

**Objective:**

To investigate older adults’ attitudes toward deprescribing by investigating which medications they would like to have deprescribed, the reasons why, and patient factors associated with interest in deprescribing.

**Design, Setting, and Participants:**

This survey study was conducted from May 2022 to December 2023 in primary care settings in 14 countries. Patients aged 65 years or older taking 5 or more medications were consecutively recruited by their general practitioner (GP) and completed the questionnaire.

**Exposures:**

Patient characteristics, including gender, number of medications, GP gender, education level, financial status, confidence in completing medical forms, self-rated health, satisfaction with medications, trust in the GP, and country.

**Main Outcomes and Measures:**

The primary outcomes were patient attitudes toward deprescribing specific medications, as measured by responses to the question, “Thinking about your current medication list, are there any medications that you would like to stop taking or reduce the dose of?” Multilevel multivariable logistic regression analysis was used, adjusted for clustering effect at the country level, to investigate the association between patient characteristics and interest in deprescribing.

**Results:**

Of 1340 patients (mean [SD], 96 [47] patients per country), 736 (55%) were women, 580 (44%) had secondary school as their highest level of education, 1089 (82%) were satisfied with their medications, and 589 (44%) expressed they would like to deprescribe 1 or more of their medications. Patients expressed interest in deprescribing specific medications at varying levels, from 79% (86 of 109 patients) in Poland to 23% (21 of 96 patients) in Bulgaria. The 3 most reported medications patients would like to have deprescribed were diuretics (111 of 1002 medications [11%]), lipid-modifying agents (109 of 1002 medications [11%]), and agents acting on the renin-angiotensin system (83 of 1002 medications [8%]). The odds of naming at least 1 specific medication for deprescribing were lower for patients with higher medication satisfaction (odds ratio, 0.31; 95% CI, 0.21-0.47) and for patients with higher trust in their GP (odds ratio, 0.960; 95% CI, 0.930-0.998).

**Conclusions and Relevance:**

In this survey study with primary care patients aged 65 years and older, patient attitudes toward deprescribing specific medications varied across countries, demonstrating that deprescribing interventions could be more impactful when adapted to specific settings and contexts. These findings highlight the importance of patient-practitioner communication in ensuring appropriate medication use.

## Key Points

**Question:**

What are older adults’ attitudes toward deprescribing specific medications?

**Findings:**

In this survey study including 1340 older adults from 14 countries, 44% expressed they would like to deprescribe 1 or more of their specific medications, with percentages varying across countries. Patients with higher medication satisfaction and higher trust in their general practitioner had significantly lower odds of wanting to deprescribe specific medications.

**Meaning:**

These findings highlight the importance of patient-practitioner communication in deprescribing and demonstrate that patient-facing intervention materials might be more impactful when adjusted to local context and different settings.

## Teaser

This survey study examines older adults’ attitudes toward deprescribing by investigating which medications they would like to have deprescribed, the reasons why, and patient factors associated with interest in deprescribing.

## Introduction

The prevalence of polypharmacy (ie, use of ≥5 medications)^1^ among older adults is high.^2,3^ Medications lacking indication, used in too high doses, or whose potential harms outweigh potential benefits are considered inappropriate.^1,4,5^ Owing to the high rates of inappropriate polypharmacy and its associated harms, interest in deprescribing (ie, stopping or reducing inappropriate medication)^6^ is increasing.^7,8^ Although overall reported patient willingness to deprescribe is high, this might vary across different settings.^9,10,11^ Patients from higher-income countries seem more willing to deprescribe.^9,10^ Variations across countries in health care systems, out-of-pocket costs, pharmaceutical marketing, and societal narratives may influence patients’ attitudes toward deprescribing.^11,12^ Therefore, it is important to understand factors associated with patient attitudes toward deprescribing.

High satisfaction with medications, perceived benefits from medications, and fear of return of symptoms have been identified as barriers to deprescribing.^13,14^ In contrast, a good patient-physician relationship has been identified as an enabler.^13,14^ Studies^10,13,15^ using the revised Patients’ Attitudes Toward Deprescribing (rPATD) questionnaire have shown that most patients report being willing to deprescribe if their physician said it was possible. Willingness was also high when patients were asked about individual medication classes.^16,17,18,19^ Patients’ reported willingness has been inconsistently associated with deprescribing in clinical practice.^15,20,21^ Nevertheless, assessing willingness may help to identify patients for medication optimization strategies.^15^ Although the rPATD has been validated and widely used in deprescribing research, it is possible that it may not capture all relevant aspects of deprescribing attitudes.^22,23^ This can be due to variations across medication classes, clinical contexts, social desirability, and the mention of whether the physician said it was possible when assessing patient attitudes.^22,23^ Variations in patient attitudes could also be related to the type of medication.^15,16,18,19,23,24,25,26^ In a vignette study,^19^ patients were more interested in deprescribing a medication used for treatment than for prevention, and in a Dutch survey study,^26^ patients were more willing to deprescribe statins than diabetic drugs. Little is known about older adults’ attitudes toward having specific medications deprescribed from their own medication list.

Shared decision-making and trust in the physician are important for successful deprescribing outcomes.^27,28,29^ Educational material for patients and decision aids may support shared decision-making by ensuring that patients are fully informed about potential benefits and harms of their medications, enabling them to make informed choices.^30^ Nevertheless, physicians may overestimate benefits of medical therapy and may have difficulty understanding and explaining deprescribing, as well as the benefits and risks of medications, to their patients.^31,32,33^ When physicians do not provide accurate information or if it is not culturally acceptable for the patient to question the physician, having trust and a good patient–general practitioner (GP) relationship may not necessarily support informed shared decision-making.^33^ Furthermore, patient values and preferences can influence both the suitability of deprescribing and their attitudes toward deprescribing, which may vary by medication class.^23,26^ Understanding patients’ attitudes toward deprescribing specific medications is important for designing effective deprescribing interventions that consider differences across medication types. In this study, we aimed to investigate older adults’ attitudes toward deprescribing across 14 countries, to assess which medications patients would like to have deprescribed and the reasons why, as well as patient factors associated with their attitudes toward deprescribing.

## Methods

### Study Design and Study Participants

This survey study was conducted in primary care settings at 17 sites in 14 countries (eFigure 1 in Supplement 1) from May 2022 to December 2023. In each country, national coordinators were responsible for recruiting 100 patients (aged ≥65 years and taking ≥5 medications regularly). Exclusion criteria were the inability to give informed consent and/or residency outside the participating countries. This study followed the American Association for Public Opinion Research (AAPOR) reporting guideline.^34^ Further details on the study design were published previously in the study protocol.^35^

The study was conducted according to the guidelines of the Declaration of Helsinki^36^ and was approved by the competent local ethics committee in Switzerland (Kantonale Ethikkommission Bern) in January 2022. This study was conducted according to the relevant regulations at the participating sites, and each national coordinator sought local ethical approval where necessary (eAppendix 1 in Supplement 1). To respect privacy rights under European regulation, requirements of the European General Data Protection Regulation were fulfilled by anonymization and data source protection. Patients gave informed consent by replying to the question, “By clicking yes here, I agree to participate in this study.” If they clicked no, they could not complete the survey.

### Data Source and Data Collection

National coordinators recruited GPs, who, in turn, consecutively recruited eligible patients (see eAppendix 2 in Supplement 1 for additional information). The study questionnaire (eAppendix 3 in Supplement 1) was anonymous and could be completed on paper or online using the REDCap survey function.^37^ The questionnaire was translated and cross-culturally adapted for each participating country by the respective national coordinators.

### Variables and Data Management

The questionnaire contained questions on patients’ sociodemographic characteristics, trust in their GP (measured by the abbreviated Wake Forest Trust in Physician Scale^38^; range, 5-25, with higher values indicating higher trust), attitudes toward deprescribing, and 2 questions from the rPATD.^39^ We selected sociodemographic characteristics on the basis of our research questions and literature.^10,40^ We considered patients who responded yes to the question, “Thinking about your current medication list, are there any medications that you would like to stop taking or reduce the dose of?” as patients wanting to deprescribe. Patients who responded yes to this question could enter the name of 1 to 4 medications they would like to stop or reduce (order did not matter) and the reasons or reasons why they chose each medication. Patients who responded no to this question could choose the reason or reasons for this from a predetermined list informed by Vordenberg et al.^19^ To classify the medications named, we used Anatomical Therapeutic Chemical codes at the second anatomical level to standardize the medication classification and group medications into specific therapeutic and pharmacological subcategories (see eAppendix 2 in Supplement 1).

### Statistical Analysis

We used descriptive statistics to report participant characteristics, frequency (number and percentage) of patients wanting to deprescribe, and reasons for wanting to or not to have any medication deprescribed. Continuous variables were presented as mean (SD) or median (IQR), and categorical variables were presented as numbers and percentages. The 3 most frequently named medications for deprescribing were stratified by patient gender and country. To study associations between patient characteristics (gender, number of medications, GP gender, financial status, confidence in completing medical forms, self-rated health, satisfaction with medications, and trust in the GP) and patients’ interest in deprescribing, we performed a multilevel multivariable logistic regression, adjusted for clustering effects at the country level. We calculated the intracluster correlation coefficient and median odds ratio (OR) in the regression model to explore the country variability in our model.^41^ We performed a sensitivity analysis using the same regression model only for countries with 60 or more patients. Considering that our outcome was not rare, we performed a sensitivity analysis using generalized estimating equations with a Poisson distribution and log link to estimate risk ratios instead of ORs, accounting for within-country correlations.^42^ We used a hypothesis-driven approach to select the covariates in the regression. A 2-sided *P* < .05 was considered statistically significant. We identified missing data at random performing the Little Missing Completely at Random test for variables with missing of 5% or more (*P* = .23) and used a complete case analysis method to handle missingness. We used Stata statistical software version 16.1 (StataCorp) to perform the analyses.^43^

## Results

There were 1423 older patients who started answering the questionnaire, of whom 1340 were included in the analysis after providing consent and/or completing more than 5 questions (including 4 eligibility questions). Among the 1340 participants, 736 (55%) were women, 580 (44%) had secondary school as the highest educational level, 716 (53%) were confident in completing medical forms, and 597 (45%) rated their health as average. Participants were taking a mean (SD) of 7 (2) regular medications, and 1089 (82%) reported being satisfied with their medications (Table 1). The number of participants per country varied from 27 in Croatia to 229 in the Netherlands (mean [SD], 96 [47] patients per country) (eFigure 1 in Supplement 1).

**Table 1.** Patient Characteristics

Characteristics^a^	Patients, No. (%) (N = 1340)
Gender^b^	
Woman	736 (55)
Man	598 (45)
Education level	
None	44 (3)
Primary school	329 (25)
Secondary school	580 (43)
Third level education	376 (28)
Ease of making ends meet	
Without any problems	340 (25)
Quite easily	451 (34)
With some difficulty	450 (34)
With great difficulty	84 (6)
Living situation	
Own house or apartment	1016 (76)
Rented house or apartment	307 (26)
Confidence in filling out medical forms	
Extremely	258 (19)
Quite a bit	458 (34)
Somewhat	340 (25)
A little bit	173 (13)
Not at all	104 (8)
Born in the country of residence	
Yes	1221 (91)
No	108 (8)
First language	
Official language of the country of residence	1252 (93)
Other language	74 (6)
Self-rated health	
Excellent	5 (1)
Very good	68 (5)
Good	462 (34)
Average	597 (45)
Poor	198 (15)
Live alone in the household	
Yes	494 (37)
No	836 (62)
No. of medications, mean (SD)	7 (2)
Medication preparation	
Self-prepare and take medication according to the prescription.	1165 (87)
Receive support in preparing and/or taking medication.	168 (13)
Trust in the GP, median (IQR)^c^	21 (19-24)
Duration of patient-GP relationship, y^d^	
0-9	617 (46)
10-19	375 (28)
20-29	178 (13)
≥30	117 (9)
GP gender^d^	
Woman	696 (52)
Man	549 (41)
Other	16 (1)^e^
GP practice location^d^	
Urban	772 (58)
Suburban	297 (22)
Rural	155 (12)

Abbreviation: GP, general practitioner.

^a^ The missing data were less than or equal to 1% for all variables, except for number of medications (3%), duration of the patient-GP relationship (4%), GP gender (6%), and GPs’ practice location (9%).

^b^ No patient chose the option *other *to the question, “What is your gender?”

^c^ Trust was measured with the score of the abbreviated Wake Forest Trust in Physician Scale^38^ (range, 5-25, with higher values indicating higher trust).

^d^ Data are shown only for 1295 participants who responded yes to the question, “Do you have your own GP/family doctor (definition: when you have a health problem, you usually consult the same family doctor, except in emergencies)?”

^e^ Eight GPs classified as other were from the Netherlands, where the patients in our sample did not have a unique fixed GP.

**Figure 1.**
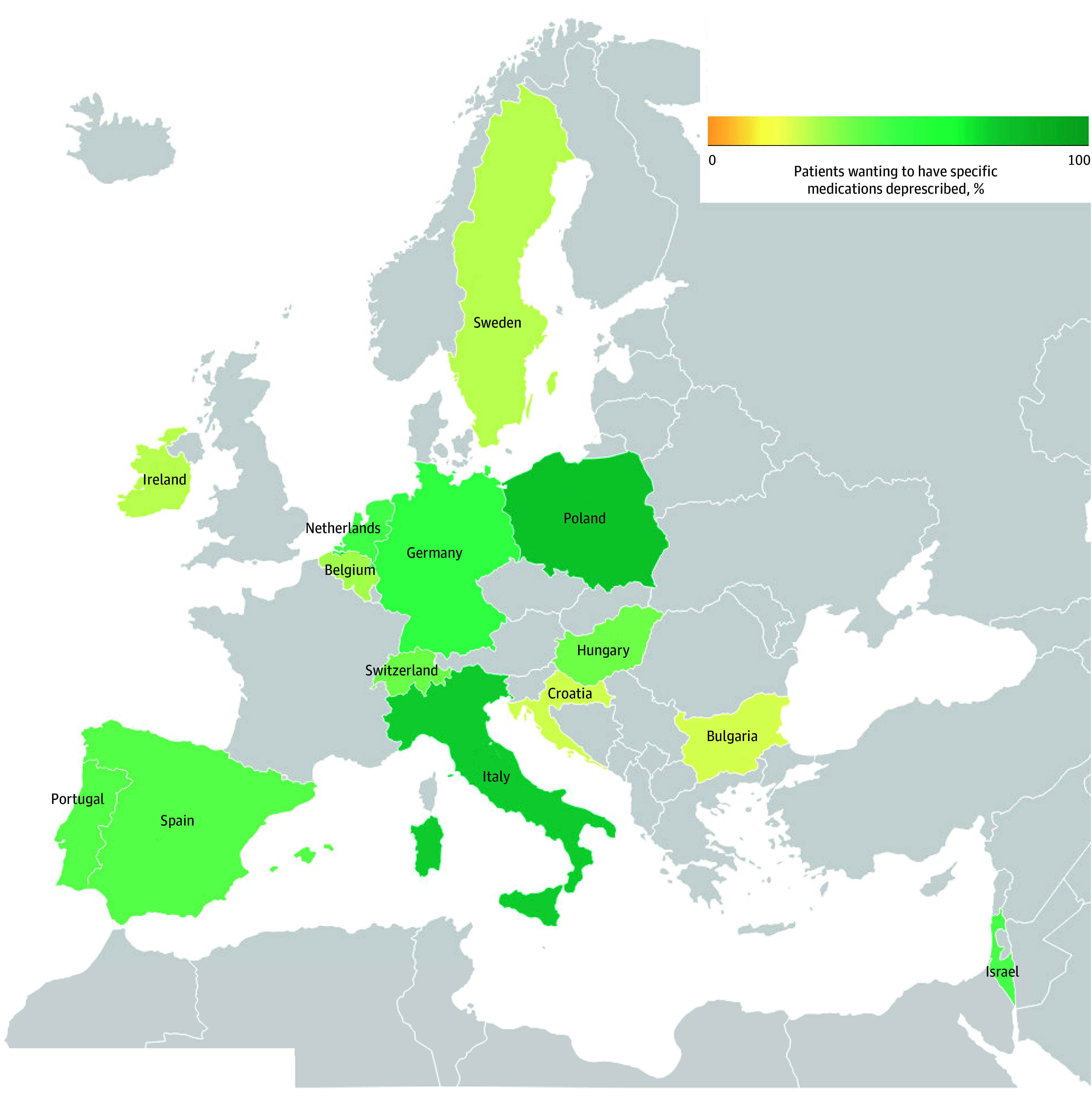
Percentage of Older Adults Per Country Who Would Like to Have at Least 1 Medication Deprescribed Participants were asked, “Thinking about your current medication list, are there any medications that you would like to stop taking or reduce the dose of?” Map was created with Datawrapper.

Regarding patients’ attitudes toward deprescribing, 1088 patients (81%) agreed or strongly agreed with the rPATD^39^ statement, “If my doctor said it was possible, I would be willing to stop one or more of my regular medicines,” 648 (48%) agreed with the statement, “I would like to try stopping one of my medicines to see how I feel without it,” and 589 (44%) responded yes to the question, “Thinking about your current medication list, are there any medications that you would like to stop taking or reduce the dose of?” (eFigure 2 in Supplement 1). Interest in deprescribing specific medications was highest among participants in Poland (86 of 109 patients [79%]) and in Italy (68 of 92 patients [75%]), and lowest among participants in Croatia (6 of 27 patients [24%]) and Bulgaria (21 of 96 patients [23%]) (Figure 1).

**Figure 2.**
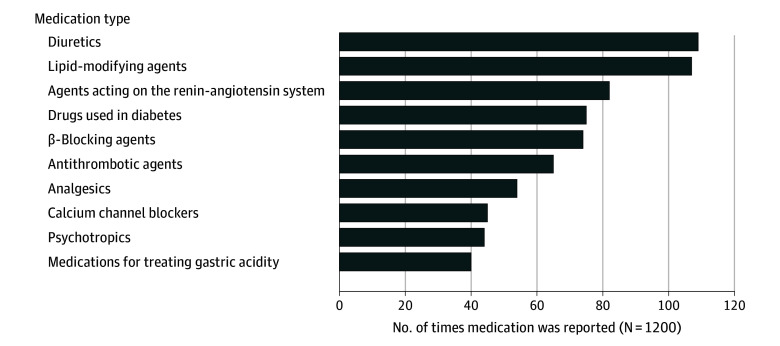
Frequency of Top 10 Medication Types That Patients Would Like to Deprescribe (n = 1002)

Patients named 1002 medications (mean [SD], 3 [3] per patient) they would like to have deprescribed, with 76 patients (6%) reporting 4 medication names. The 3 most mentioned were diuretics (111 of 1002 medications [11%]), lipid-modifying agents (109 of 1002 medications [11%]), and agents acting on the renin-angiotensin system (83 of 1002 medications [8%]) (Figure 2). Psychotropics (44 of 1002 medications [4%]) and medications used for treating gastric acidity (40 of 1002 medications [4%]) were also often named (Figure 2). Diuretics were among the most named medications for both women (63 of 577 patients [11%]) and men (45 of 425 patients [11%]), but when stratifying the analysis by country, diuretics were the top 1 only for Italy (20 of 157 patients [13%]) and Poland (54 of 263 patients [21%]) (eTable 1 in Supplement 1).

**Figure 3.**
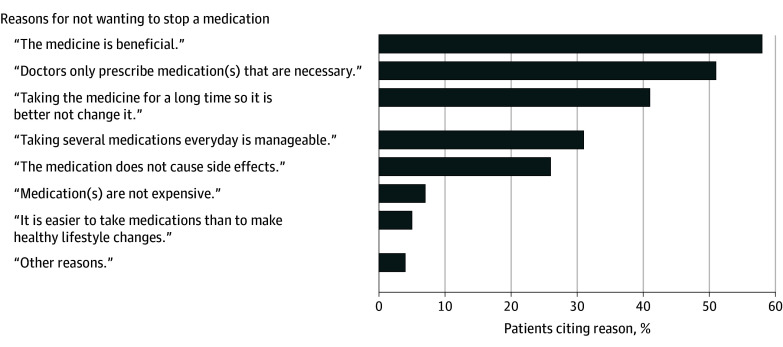
Participants’ Reasons for Not Wanting a Medication Deprescribed (n = 726) Participants could choose multiple responses.

The most reported reasons for wanting to deprescribe were presence of adverse effects associated with the medication (271 of 589 patients [46%]), dislike of medication (144 of 589 patients [24%]), and inconvenience of taking the medication (131 of 589 patients [22%]). The presence of adverse effects was also the most reported reason for wanting to deprescribe any of the top 5 medications (eFigure 3 in Supplement 1). Patients who did not want to deprescribe any of their medications identified reasons for reluctance, including medication benefits (422 of 726 patients [58%]), belief that physicians only prescribe necessary medications (366 of 726 patients [50%]), and habit of taking the medication for a long time (294 of 726 patients [41%]) (Figure 3 and eTable 2 in Supplement 1).

Patients with higher satisfaction with medications (adjusted OR, 0.31; 95% CI, 0.21-0.47) and higher trust in their GP (adjusted OR, 0.960; 95% CI 0.930-0.998) had lower odds of wanting to deprescribe specific medications (Table 2). In the unadjusted analysis, the odds of wanting to deprescribe were higher with higher number of medications (OR, 1.05; 95% CI, 1.00-1.11). The odds of wanting to deprescribe varied significantly between countries, with the median odds of patients wanting to deprescribe differing by a factor of 2.27 between 2 randomly selected countries (median OR, 2.27; 95% CI, 1.08-3.19). Approximately 10.2% of the variance in patients wanting to deprescribe can be attributed to differences between countries (intracluster correlation coefficient, 0.10; 95% CI, 0.04-0.22). Sensitivity analysis considering only countries with 60 or more patients showed similar results (eTable 3 in Supplement 1). In sensitivity analysis estimating relative risks, we identified patients with higher medication satisfaction to be less likely to want to deprescribe (relative risk, 0.64; 95% CI, 0.51-0.80), and the direction of the association between trust in the GP and the outcome remained the same, but became not statistically significant (relative risk, 0.98; 95% CI, 0.96-1.01) (eTable 4 in Supplement 1).

**Table 2.** Association Between Interest in Deprescribing Any Specific Medication and Sociodemographic Characteristics (n = 1081)^a^

Variable	Crude OR (95% CI)	*P* value	Adjusted OR (95% CI)	*P* value^b^
Patient gender (reference, man)				
Woman	1.08 (0.85-1.36)	.52	1.12 (0.86-1.50)	.40
No. of medications per unit increase	1.05 (1.00-1.11)	.04	1.05 (0.99-1.12)	.11
GP gender (reference, woman)				
Man	1.24 (0.97-1.60)	.09	1.27 (0.96-1.69)	.10
Other	1.05 (0.35-3.15)	.93	1.25 (0.33-4.69)	.75
How do you make ends financially? (reference, with great difficulty)				
Without any problems	0.84 (0.49-1.44)	.52	0.83 (0.44-1.55)	.55
Quite easily	0.66 (0.39-1.10)	.11	0.66 (0.36-1.22)	.18
With some difficulty	0.94 (0.56-1.55)	.79	0.94 (0.53-1.68)	.85
How confident are you filling out medical forms by yourself? (reference, not at all)				
Extremely	0.82 (0.49-1.37)	.45	1.26 (0.69-2.30)	.45
Quite a bit	1.00 (0.63-1.61)	.99	1.55 (0.88-2.72)	.13
Somewhat	0.99 (0.61-1.60)	.96	1.35 (0.76-2.39)	.30
A little bit	0.82 (0.47-1.42)	.48	1.17 (0.62-2.20)	.63
Self-rated health (reference not good health state)^c^				
Good health state	0.80 (0.63-1.02)	.08	1.00 (0.74-1.34)	>.99
Overall, I am satisfied with my current medications (reference, no)^d^				
Yes	0.29 (0.20-0.40)	<.001	0.31 (0.21-0.47)	<.001
Trust in the GP, per unit increase^e^	0.95 (0.92-0.98)	<.001	0.960 (0.930-0.998)	.04
Intracluster correlation coefficient	NA	NA	0.10 (0.04-0.22)	NA
Median value of the OR	NA	NA	2.27 (1.08-3.19)	NA

Abbreviations: GP, general practitioner; NA, not applicable; OR, odds ratio.

^a^ Patients who responded yes to the question, “Thinking about your current medication list, are there any medications that you would like to stop taking or reduce the dose of?” were considered to want to deprescribe specific medications.

^b^ Mixed-models logistic regression was adjusted at the country level. The dependent variable was wanting to deprescribe.

^c^ Self-rated health was dichotomized, considering good, very good, and excellent as good health state, and average and poor as not good health state.

^d^ Satisfaction with currently medication was assessed by the 5-point Likert scale question, “Overall, I am satisfied with my current medications,” from Reeve et al.^39^ Responses to the 5-point Likert scale question were dichotomized. Responses of agree or strongly agree were considered as yes.

^e^ Determined by score of the abbreviated Wake Forest Trust in Physician Scale^38^ (range, 5-25, with higher values indicating higher trust).

## Discussion

In this international survey study with 1340 participants from 14 different countries, nearly one-half of older adults with polypharmacy would like to stop or reduce at least 1 of their mean of 7 medications. Attitudes toward deprescribing differed across countries. Medications used for cardiovascular diseases were the most named for deprescribing, because of adverse effects. Lower satisfaction with medications and trust in the GP were associated with wanting to deprescribe specific medications.

We observed geographic variation in our findings, with Poland and Italy having the highest proportion of patients who would like to deprescribe, and Bulgaria and Croatia the lowest. This is in line with reported variations in patients’ willingness to deprescribe across French-speaking countries, including Belgium, Canada (province of Quebec), France, and Switzerland’s French-speaking region.^44^ Such variations may be due to differences in health literacy, income, health care systems, and out-of-pocket spending on medication.^9,11,45^ Higher-income countries often have higher health literacy, a better understanding of medications, and more initiatives to optimize medications than low-income countries.^9,11^ A systematic review and meta-analysis^9^ found that willingness to deprescribe varied across countries and seemed to be higher in patients from higher-income countries (eg, UK, Netherlands, and Italy). However, that review was limited by comparing findings from studies with different ways of data collection. Context-specific differences should be considered when designing and implementing deprescribing interventions (eg, when running multinational deprescribing trials or when using deprescribing materials developed in other countries in research or clinical practice).

The medications most frequently named by older adults with polypharmacy for deprescribing were those usually used in the treatment or prevention of cardiovascular diseases (diuretics, lipid-modifying agents, and agents acting on the renin-angiotensin system). This is in line with findings from a survey study^46^ conducted in the US involving adults aged 50 to 80 years and with a Dutch study^26^ in which patients were willing to stop their cardiovascular medications. The presence of adverse effects was the most common reason for wanting to have these medications deprescribed, in line with other studies.^47^ Increased frequency of urination is part of the mode of action of diuretics, which is seen as an unfavorable adverse effect by patients and can greatly affect their quality of life.^48^ When adverse effects are noticed before benefits for preventative medications, it is understandable that patients would like to discontinue these medications.^19^ Furthermore, for this type of medication, the benefits are often only visible in the long term or are not visible at all (ie, through absence of cardiovascular events), and the effects are observed in clinical tests rather than patients’ perceptions. Hence, patients may be more likely to want to stop medications for which they do not perceive an effect rather than those prescribed for symptomatic health issues.^19^ This finding may also indicate that patients may not always be informed about the reasons for taking medications, highlighting the importance of explaining the benefits and risks of medications when involving patients in deprescribing decisions. This proposed rationale aligns with the most frequently reported reasons for wanting to deprescribe identified in this research: presence of adverse effects, dislike of medication, and inconvenience of taking the medication.^47^ However, cardiovascular medications are often used by older adults, which could have contributed to their being among the most named for deprescribing. Nevertheless, cardiovascular medications are also deprescribing targets when inappropriate.^49^

Not all forms of polypharmacy merit equal attention. Although cardiovascular medications are not so often inappropriate given their adverse effects, psychotropics are often a priority for deprescribing.^25,50^ Interestingly, psychotropic medications were also among the 10 most reported medications patients would like to deprescribe, although other studies^51,52^ have reported that patients may be reluctant to deprescribe these medications. The long-term benefits of psychotropic medications are questionable, and it is possible that patients would like to deprescribe these medications because they perceive their adverse effects but not their benefits. Given the risks associated with psychotropic medications, such as drug-drug or drug-disease interaction and physiological dependence, these medications are potential deprescribing targets. This combined with patients’ interest in deprescribing these medications suggests that deprescribing interventions targeting psychotropic drugs may be particularly successful.

The most frequent reasons for older adults not wanting to deprescribe were the benefits associated with using medications, in line with studies reporting favorable perceptions of medications as a barrier to deprescribing.^13,14,53^ One common reason for not wanting to deprescribe was the belief that physicians only prescribe necessary medications. This highlights the importance of the patient-practitioner relationship, trust in the GP, physician education on medication optimization, and communication that the benefits and harms of medications can change over time.^19^ Older adults with higher satisfaction with medication and higher trust in their GP tended to be less likely to want to deprescribe in our study. Indeed, low satisfaction with medications has been reported as an enabler to deprescribing.^13,14^ As expected, patients satisfied with their medications have no incentive to change them. Furthermore, patients who trust their GPs may be more satisfied with their overall care and less likely to challenge their GPs’ medication decisions.^54^ Nevertheless, the association between trust and wanting to deprescribe was tenuous, and although some studies reported trust in the physician as an enabler for deprescribing, others did not find an association.^13,14,53,55^ This difference could be explained by the fact that we based our analysis on a question asking whether patients would like to deprescribe any of their own specific medications, whereas previous studies examined patients’ willingness to deprescribe if their physician said it was possible, but this still requires confirmatory research.^23^

The overall willingness to deprescribe in our study was 81% using the global question from the rPATD, “If my doctor said it was possible, I would be willing to stop one or more of my regular medicines,” and 48% using the question, “I would like to try stopping one of my medicines to see how I feel without it,” in line with studies that identified the same differences using the same questions.^10,20^ In addition, 44% of the participants reported wanting to deprescribe 1 or more specific medications they were using. On the basis of the current evidence on patients’ reported attitudes toward deprescribing and actual behavior in implementing deprescribing decisions in clinical practice being mixed,^15,20,21^ we cannot expect that patients’ interest in deprescribing automatically translates into successful deprescribing. Many patients refuse to participate in deprescribing trials and refuse real-world deprescribing suggestions.^15,25^ It is possible that not naming medications usually suitable for deprescribing is related to the low willingness to participate in deprescribing trials focusing on such medications.^25,50^ Nevertheless, psychotropics are often deprescribing targets and were often named by older adults in our study.^25,50^ To engage patients in deprescribing, a patient-centered approach is essential, involving shared decision-making, communication about risks and benefits, and addressing patient fears and concerns.

This study is strengthened by its novel aspect of investigating specific medications patients would like to have deprescribed and the reasons why. Another strength is its international design involving 17 sites from 14 countries, which allowed us to compare patients’ attitudes toward deprescribing across countries, even more of them than initially planned.^35^ We were also able to explore reasons why patients would not like to deprescribe and important contextual factors such as health literacy, socioeconomic status, and trust in the GP.

### Limitations

This study also has limitations. First, since we did not have access to patients’ complete medication lists, we were unable to validate self-reported medication use and adjust the analysis for medication types used. Second, since we did not have information about patients’ diagnoses and health status, we were unable to assess the appropriateness of deprescribing preferences. Because of the hypothetical nature of the deprescribing questions, the reported deprescribing attitudes may not reflect patients’ behaviors in real-world situations. For feasibility reasons, patients could only name a maximum of 4 medications for deprescribing. When piloting the questionnaire, no participant indicated more than 4 medications and only 6% of the study respondents reported 4 medications. Owing to the anonymous data collection via national coordinators and GPs in different countries, we were unable to track response rates. The fact that (except for the Netherlands) samples were not random, and the overall high health literacy, high socioeconomic level, good self-rated health, and low representativeness for immigrants limit the overall generalizability of our findings and their representativeness for populations such as those with immigrant status, low socioeconomic status, frail patients, and those with cognitive impairment. Although GPs were instructed to recruit patients consecutively to have a representative patient sample, we cannot rule out selection bias. If GPs recruited patients who were more open to medication optimization, selection bias may have led to an overestimation of patients’ interest in deprescribing. It is possible that GPs selected patients with whom they have a good relationship, which could have led these patients to rate their trust in their GP higher than the typical patient, and the patient-practitioner relationship may have influenced study participation. Nevertheless, having GPs as recruiters was a feasible approach, considering that GPs have access to patient information regarding the inclusion criteria (age and number of medications) and could, therefore, select and approach eligible patients during consultations. We did not collect the exact age of the patients and only asked whether they were aged 65 years or older, so we were unable to adjust the analysis for patient age.

## Conclusions

The results from this study are informative for the development of future deprescribing interventions. The observed variation in patients wanting to deprescribe across countries demonstrates that patient-facing intervention materials might be more impactful when adjusted to local context. When designing future deprescribing interventions, the types of medications patients would like to deprescribe should be considered and weighted in conjunction with the prescriber’s expertise and guidelines. Medications whose benefits are challenging to estimate in older adults, such as preventative agents (eg, lipid-modifying agents) and psychotropics, were often named for deprescribing. Educational material for patients and decision aids may support shared decision-making, especially when the benefits of certain treatments are uncertain. The association between patients’ satisfaction with medications, trust in their GP, and interest in deprescribing specific medications highlights the importance of patient-practitioner communication in deprescribing. Future research should aim at better understanding the relationship between trust and patient-practitioner relationships and how this influences the implementation of deprescribing decisions in real-world clinical settings.

## Notes

Supplement 1. **eAppendix 1.** Additional information by ethical approval by study site **eFigure 1.** Recruitment flow chart **eFigure 2.** Patients’ willingness to stop or reduce medications (n=1,340) **eTable 1.** Patients’ attitudes towards deprescribe: Most frequently mentioned medication classes, stratified by patient gender and country (n=1,002) **eFigure 3.** Reasons for willingness to stop or reduce medications according to the medication class of the three most mentioned medication classes (n=589) **eTable 2.** Other reasons for older patients not wanting to have a medication deprescribed (n=29) **eTable 3.** Sensitivity analysis with countries with ≥60 patients: Association between interest in deprescribing any specific medication and sociodemographic characteristics (n=1,037) **eTable 4.** Sensitivity analysis: Association between interest in deprescribing any specific medication and sociodemographic characteristics using Generalized Estimating Equations (GEE) (n=1,081) **eAppendix 2.** Additional information
eReferences
**eAppendix 3.** Study questionnaire

Supplement 2.
Data Sharing Statement
