# Supplementary material for: Older Adults’ Attitudes Toward Deprescribing in 14 Countries
Source: JAMA Netw Open. 2025 Feb 10;8(2):e2457498. doi: 10.1001/jamanetworkopen.2024.57498 (PMC11811803; doi:10.1001/jamanetworkopen.2024.57498)
Supplement: Supplement 1. — eAppendix 1. Additional information by ethical approval by study site eFigure 1. Recruitment flow chart eFigure 2. Patients’ willingness to stop or reduce medications (n=1,340) eTable 1. Patients’ attitudes towards deprescribe: Most frequently mentioned medication classes, stratified by patient gender and country (n=1,002) eFigure 3. Reasons for willingness to stop or reduce medications according to the medication class of the three most mentioned medication classes (n=589) eTable 2. Other reasons for older patients not wanting to have a medication deprescribed (n=29) eTable 3. Sensitivity analysis with countries with ≥60 patients: Association between interest in deprescribing any specific medication and sociodemographic characteristics (n=1,037) eTable 4. Sensitivity analysis: Association between interest in deprescribing any specific medication and sociodemographic characteristics using Generalized Estimating Equations (GEE) (n=1,081) eAppendix 2. Additional information eReferences eAppendix 3. Study questionnaire [file jamanetwopen-e2457498-s001.pdf]

Supplemental Online Content

Vidonscky Lüthold R, Jungo KT, Weir KR, et al. Older adults' attitudes toward deprescribing in 14 countries. *JAMA Netw Open*. 2025;8(2):e2457498.
doi:10.1001/jamanetworkopen.2024.57498

eAppendix 1. Additional information by ethical approval by study site

eFigure 1. Recruitment flow chart

eFigure 2. Patients' willingness to stop or reduce medications (n=1,340)

eTable 1. Patients' attitudes towards deprescribe: Most frequently mentioned medication classes, stratified by patient gender and country (n=1,002)

eFigure 3. Reasons for willingness to stop or reduce medications according to the medication class of the three most mentioned medication classes (n=589)

eTable 2. Other reasons for older patients not wanting to have a medication deprescribed (n=29)

eTable 3. Sensitivity analysis with countries with ≥60 patients: Association between interest in deprescribing any specific medication and sociodemographic characteristics (n=1,037)

eTable 4. Sensitivity analysis: Association between interest in deprescribing any specific medication and sociodemographic characteristics using Generalized Estimating Equations (GEE) (n=1,081)

eAppendix 2. Additional information

eReferences

eAppendix 3. Study questionnaire

This supplemental material has been provided by the authors to give readers additional information about their work.

eAppendix 1. Additional information by ethical approval by study site.

The data collection in Spain was authorised by Hospital Universitario12 de Octobre (Project 23/193). In Ireland, the Social Research and Ethics Committee of University College Cork approved the ethics application (Log number 2023-135). In Israel, the study was approved by Maccabi Healthcare Services (MHS') IRB, number: 0007-23-MHS. This study was authorised by the ethics committee from Belgium (Project-ID 2022/172), Bulgaria (Project-ID 2023_RKNE_0986BCB6DF_reques), Croatia (Project-ID 251-510-03-20-2301), Germany (Project-ID 10473_BO_K_2022), Hungary (Project-ID BM/15411-3/2023), Italy (waived), Netherlands (Project_ID 22-3019), Poland (waived), Portugal (Project-ID 53/2023)), and Sweden (project number 2022-01876-01).

© 2025 Vidonscky Lüthold R et al. *JAMA Network Open*.

eFigure 1. Recruitment flow chart.

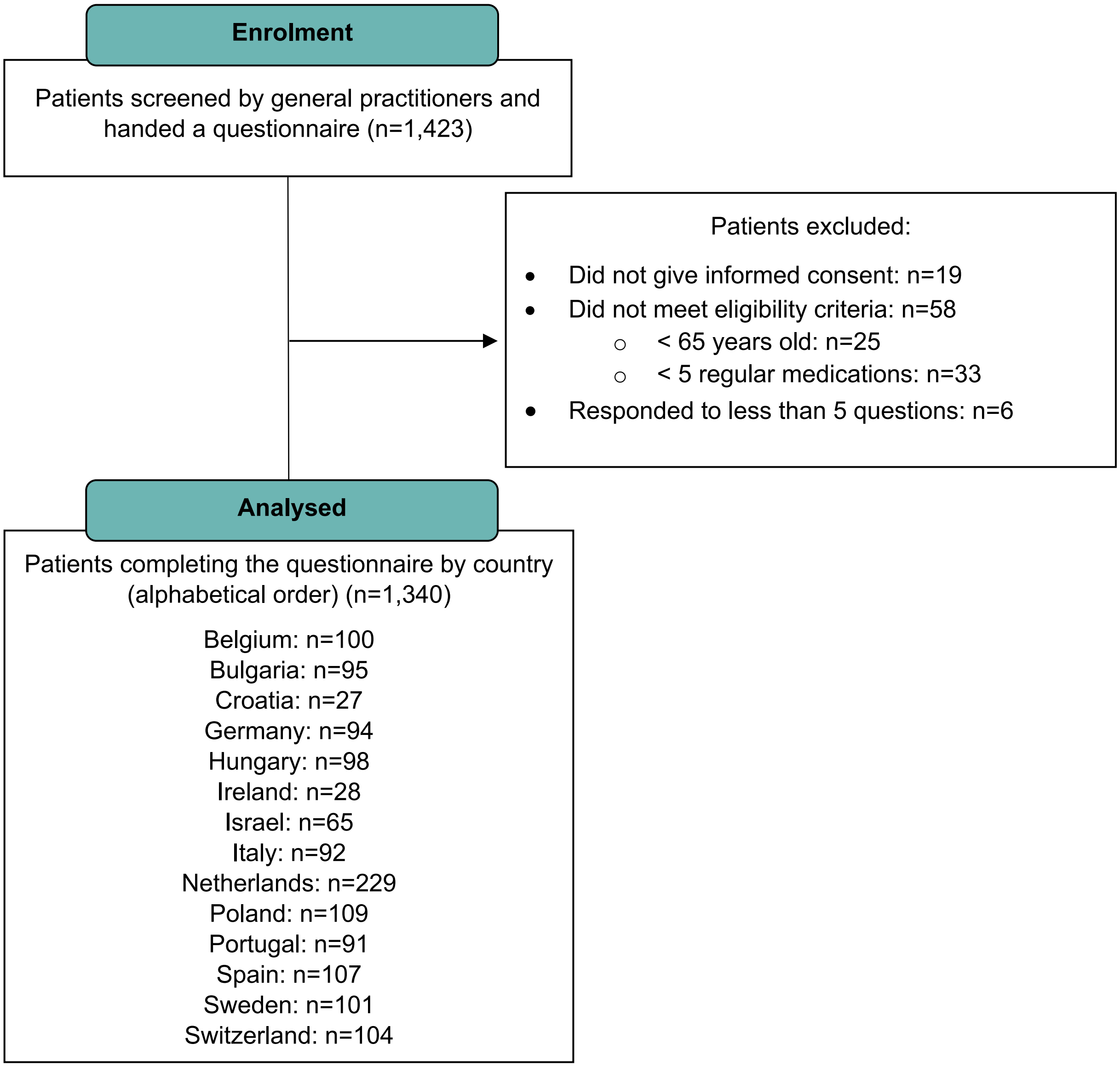

© 2025 Vidonscky Lüthold R et al. *JAMA Network Open*.

eFigure 2. Patients' willingness to stop or reduce medications (n=1,340).

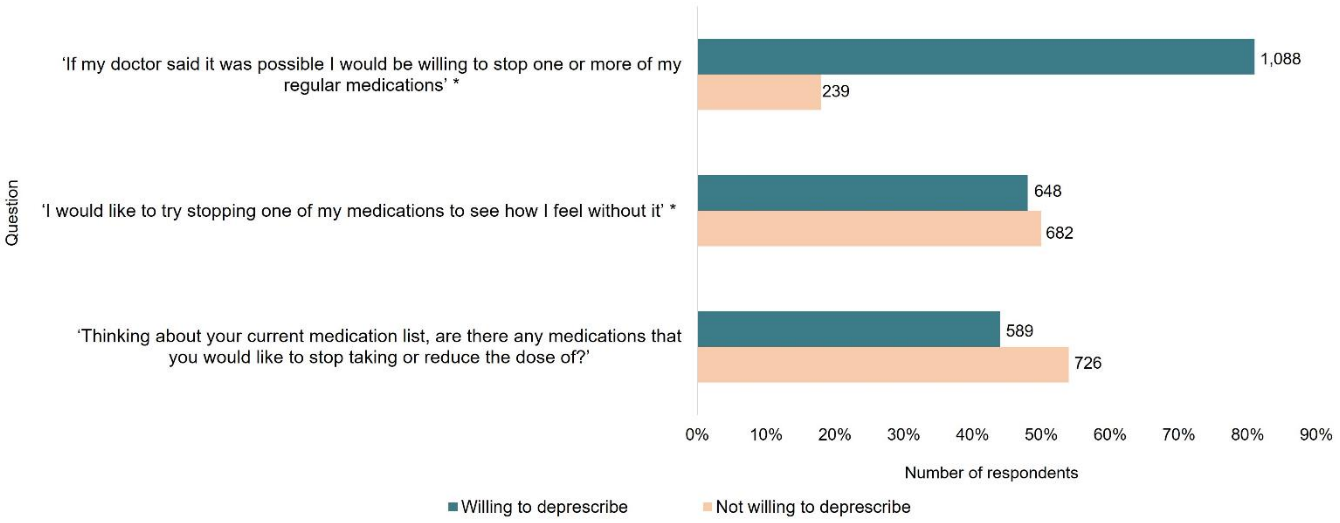

*From the revised Patients' attitudes towards deprescribing (rPATD) questionnaire.[1]
Patients who responded 'agree' or 'strongly agree' to the rPATD global questions *'If my doctor said it was possible, I would be willing to stop one or more of my regular medications'* and *'I would like to try stopping one of my medications to see how I feel without it*' were considered as willing to deprescribe. Patients who responded 'yes' to the question *'Thinking about your current medication list, are there any medications that you would like to stop taking or reduce the dose of?'* were considered willing to deprescribe.

© 2025 Vidonscky Lüthold R et al. *JAMA Network Open*.

eTable 1. Patients' attitudes towards deprescribe: Most frequently mentioned medication classes, stratified by patient gender and country (n=1,002).

	1st medication class	2nd medication class	3rd medication class
**Overall** (n=1,002 medications)	diuretics (n=109)	lipid modifying agents (n=107)	agents acting on the renin-angiotensin system (n=82)
**Gender**			
Woman (n=577)	diuretics (n=63)	lipid modifying agents (n=61)	agents acting on the renin-angiotensin system (n=46)
Man (n=425)	diuretics (n=46)	lipid modifying agents (n=46)	antithrombotic agents (n=39)
**Country**			
Belgium (n=14)	drugs used in diabetes (n=3)	psycholeptics (n=3)	antithrombotic agents (n=2)
Bulgaria (n=21)	lipid modifying agents (n=4)	diuretics (n=3)	psychotropic (n=3)
Croatia (n=10)	psycholeptics (n=4)	analgesics (n=2)	drugs used in diabetes (n=1)
Germany (n=60)	lipid modifying agents (n=11)	antithrombotic agents (n=7)	agents acting on the renin-angiotensin syst. (n=7)
Hungary (n=51)	lipid modifying agents (n=7)	drugs used in diabetes (n=6)	agents acting on the renin-angiotensin syst. (n=4)
Ireland (n=10)	agents acting on the renin-angiotensin system (n=2)	lipid modifying agents (n=2)	psychotropic (n=2)
Israel (n=33)	antithrombotic agents (n=7)	psychotropic (n=4)	medications for treating gastric acidity (n=3)
Italy (n=157)	diuretics (n=20)	antithrombotic agents (n=13)	drugs for obstructive airway diseases (n=12)
Netherlands (n=170)	agents acting on the renin-angiotensin system (n=22)	lipid modifying agents (n=22)	medications for treating gastric acidity (n=20)
Poland (n=263)	diuretics (n=54)	beta blocking agents (n=42)	agents acting on the renin-angiotensin system (n=29)
Portugal (n=49)	lipid modifying agents (n=9)	antithrombotic agents (n=5)	diuretics (n=5)
Spain (n = 60)	analgesics (n=8)	drugs used in diabetes (n=7)	lipid modifying agents (n=5)
Sweden (n=46)	drugs used in diabetes (n=7)	lipid modifying agents (n=5)	antithrombotic agents (n=4)
Switzerland (n=58)	agents acting on the renin-angiotensin syst. (n=7)	lipid modifying agents (n=7)	analgesics (n=7)

Medication classes were defined using the Anatomical Therapeutic Chemical Classification (ATC) at the second anatomical level.

© 2025 Vidonscky Lüthold R et al. *JAMA Network Open*.

eFigure 3. Reasons for willingness to stop or reduce medications according to the medication class of the three most mentioned medication classes. Multiple responses possible (n=589).

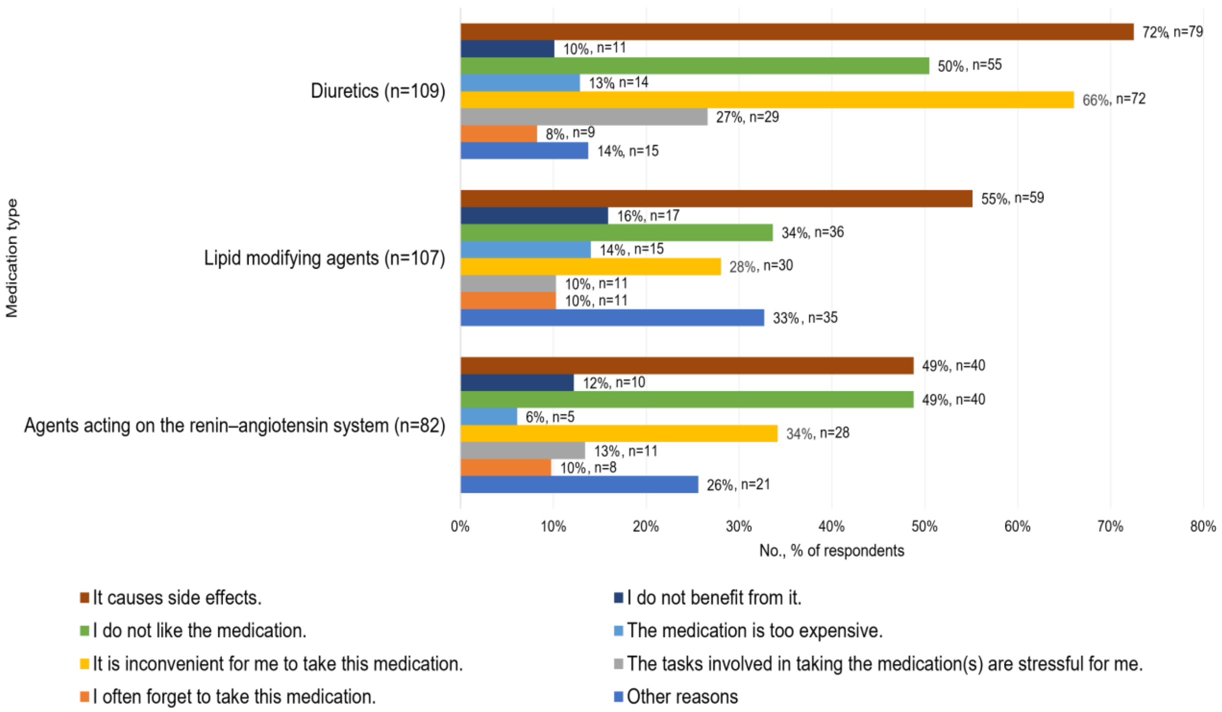

© 2025 Vidonscky Lüthold R et al. *JAMA Network Open*.

eTable 2. Other reasons for older patients not wanting to have a medication deprescribed (n=29)

**1. Trust in Healthcare Providers**
Examples:
"I only want to stop or reduce under the supervision of my (family) doctor."
"I think the doctor should decide all this, I am not a doctor, but I am open to it if possible."
**2. Fear of Adverse Effects or Recurrence of Illness**
Examples:
"Fear of feeling unwell."
"They have stopped it before, and I get sick after stopping it."
**3. Chronic Condition Management**
Examples:
"With COPD, stopping medication is not possible."
"My medications guarantee my survival."
**4. Medication Perceived as Indispensable**
Examples:
"The medications I take are all, in principle, indispensable."
"Each medicine is for a different disease."
**5. Lack of Knowledge or Lack of Opportunities**
Examples:
"You always talk about doctors. Most of the medications are prescribed by other specialists."
"I have a different doctor every time, no one I can discuss my medications with."
**6. Positive Perception of Current Treatment**
Examples:
"I feel good with it."

Free translation of the free-text responses.

eTable 3. Sensitivity analysis with countries with ≥60 patients: Association between interest in deprescribing any specific medication[#] and sociodemographic characteristics (n=1,037).

	Adjusted Odds Ratio (95% CI)	p-value [a]
**Patient gender** (ref. man)		
Woman	1.12 (0.85 to 1.47)	0.42
**Number of medications**		
per unit increase	1.06 (0.99 to 1.13)	0.07
**GP gender** (ref. woman)		
Man	1.31 (0.98 to 1.75)	0.07
Other	1.25 (0.33 to 4.73)	0.75
'How do you make ends financially?' (ref. With great difficulty)		
Without any problems	0.78 (0.41 to 1.47)	0.44
Quite easily	0.62 (0.34 to 1.15)	0.13
With some difficulty	0.90 (0.50 to 1.60)	0.71
'How confident are you filling out medical forms by yourself?' (ref. not at all)		
Extremely	1.37 (0.73 to 2.55)	0.32
Quite a bit	1.64 (0.92 to 2.93)	0.10
Somewhat	1.38 (0.77 to 2.48)	0.29
A little bit	1.38 (0.72 to 2.64)	0.33
Self-rated health [b] (ref. not good health state)		
Good health state	0.16 (0.77 to 1.42)	0.77
'Overall, I am satisfied with my current medications' [c] (ref. no)		
Yes	0.06 (0.21 to 0.47)	**0.00**
**Trust in the GP** [d]		
per unit increase	0.02 (0.21 to 0.46)	**0.06**
**ICC**	0.97 (0.04 to 0.22)	NA
**MOR**	2.57 (1.24 to 2.51)	NA

ICC: intra cluster correlation. MOR: median value of the odds ratio

[#] Patients who responded *'yes'* to the question '*Thinking about your current medication list, are there any medications that you would like to stop taking or reduce the dose of?'.* were considered to want to deprescribe specific medications.

[a] Mixed-models logistic regression adjusted at the county level. Dependent variable: wanting to deprescribe[#].

[b] Self-rated health was dichotomised considering '*good*', '*very good'* and *'excellent'* as '*good health state'* and *'average'* and *'poor'* as 'not *good health state'*.

[c] Satisfaction with currently medication was assessed by the 5-point Likert scale question *'Overall, I am satisfied with my current medications.'* from Reeve et al., 2016 [1]. 5-point Likert scale question was dichotomised. Responses '*agree*' or '*strongly agree'* were considered as '*yes*'.

[d] Score of the abbreviated Wake Forest Trust in Physician Scale [2]. Score is within 5 to 25, with higher values indicating higher trust.

© 2025 Vidonscky Lüthold R et al. *JAMA Network Open*.

eTable 4. Sensitivity analysis: Association between interest in deprescribing any specific medication[#] and sociodemographic characteristics using Generalized Estimating Equations (GEE) (n=1,081).

	Relative Risk (95% CI)	p-value [a]
**Patient gender** (ref. man)		
Woman	1.05 (0.88 to 1.26)	0.58
**Number of medications**		
per unit increase	1.02 (0.99 to 1.06)	0.23
**GP gender** (ref. woman)		
Man	1.12 (0.93 to 1.35)	0.22
Other	1.11 (0.45 to 2.76)	0.82
'How do you make ends financially?' (ref. With great difficulty)		
Without any problems	0.92 (0.62 to 1.38)	0.70
Quite easily	0.83 (0.56 to 1.21)	0.13
With some difficulty	0.97 (0.68 to 1.39)	0.89
'How confident are you filling out medical forms by yourself?' (ref. not at all)		
Extremely	1.11 (0.75 to 1.65)	0.60
Quite a bit	1.24 (0.86 to 1.79)	0.25
Somewhat	1.15 (0.79 to 1.66)	0.46
A little bit	1.06 (0.71 to 1.59)	0.77
Self-rated health [b] (ref. not good health state)		
Good health state	1.00 (0.81 to 1.22)	0.96
'Overall, I am satisfied with my current medications' [c] (ref. no)		
Yes	0.64 (0.51 to 0.80)	**0.00**
**Trust in the GP** [d]		
per unit increase	0.98 (0.96 to 1.01)	0.13

Generalized Estimating Equations (GEE), accounting for within-country correlations and specifying a Poisson regression model.

[#] Patients who responded *'yes'* to the question '*Thinking about your current medication list, are there any medications that you would like to stop taking or reduce the dose of?'.* were considered to want to deprescribe specific medications.

[a] Mixed-models logistic regression adjusted at the county level. Dependent variable: wanting to deprescribe[#].

[b] Self-rated health was dichotomised considering '*good*', '*very good'* and *'excellent'* as '*good health state'* and *'average'* and *'poor'* as 'not *good health state'*.

[c] Satisfaction with currently medication was assessed by the 5-point Likert scale question *'Overall, I am satisfied with my current medications.'* from Reeve et al., 2016 [1]. 5-point Likert scale question was dichotomised. Responses '*agree*' or '*strongly agree'* were considered as '*yes*'.

[d] Score of the abbreviated Wake Forest Trust in Physician Scale [2]. Score is within 5 to 25, with higher values indicating higher trust.

© 2025 Vidonscky Lüthold R et al. *JAMA Network Open*.

eAppendix 2: Additional information

A. Additional information about the questionnaire

The questionnaire contained questions on patients' socio-demographic characteristics, trust in their GP, and attitudes towards deprescribing. Patients' interest in having specific medications deprescribed was assessed through the binary question '*Thinking about your current medication list, are there any medications that you would like to stop taking or reduce the dose of?*'. In addition, to assess patient attitudes towards deprescribe in different ways, we included three questions from the rPATD questionnaire about patients' attitudes towards deprescribing: '*If my doctor said it was possible, I would be willing to stop one or more of my regular medicines'*, *'Overall, I'm satisfied with my current medicines'* and *'I would like to try stopping one of my medicines to see how I feel without it*'.[1] The questionnaire was piloted with seven older adults. Questionnaires from all sites were appended and analysed together.

B. Additional information about medication information data cleaning across sites

Patients named the specific medications they would like to deprescribe using brand or substance names. To classify the medications that patients would like to have deprescribed, we used ATC codes at the second anatomical level to standardize the medication classification, which allowed us to group medications into specific therapeutic and pharmacological subcategories. For instance, within the anatomical group 'C Cardiovascular System,' the second-level ATC codes can include subdivisions like 'C01 Antiarrhythmics,' 'C02 Antihypertensives,' and 'C03 Diuretics'. Since medication information was collected in different countries in different languages, we translated the medication names to German and classified the medications using the list with ATC codes from the Swiss Federal Office of Public Health.[3] For medications that were not on the list, the ATC codes were added based on information provided on the World Health Organization (WHO) website.[4] All data cleaning was done centrally by the team at the University of Bern.

eReferences

1. Reeve E, Low LF, Shakib S, Hilmer SN. Development and Validation of the Revised Patients' Attitudes Towards Deprescribing (rPATD) Questionnaire: Versions for Older Adults and Caregivers. *Drugs Aging.* 2016;33(12):913-928.
2. Dugan E, Trachtenberg F, Hall MA. Development of abbreviated measures to assess patient trust in a physician, a health insurer, and the medical profession. *BMC Health Serv Res.* 2005;5:64.
3. Spezialitätenliste (SL). Available at https://www.xn--spezialittenliste-yqb.ch/default.aspx.
4. WHO Collaborating Centre for Drug Statistic Methodology, 2023. ATC/DDD Index 2023. Oslo, Norway. http://www.whocc.no/ATC_DDD_INDEX.

© 2025 Vidonscky Lüthold R et al. *JAMA Network Open*.

eAppendix 3. Study questionnaire

Dear Madam or Sir,

Thank you for your interest in participating in this study. It is being conducted by the Institute of Primary Health Care (BIHAM) of the University of Bern with collaborators from various European countries.

You were informed about this study in your GP's practice and received this survey because your GP thinks you fulfill the inclusion criteria. We are conducting this study with adults who are 65 years or older and regularly take 5 or more medications. The aim of this project is to find out your opinion about stopping or reducing the dose of your medication. This is a survey only and no changes will be made to your medication as part of this project. The study ends after you have completed the survey.

In total, about 1000 patients from 14 countries are taking part in this survey. By taking part in this study, you contribute to better understanding how this group (adults aged 65 years or older, taking five or more medicines) think and feel about their medication. The results, which are based on the views of all the patients surveyed in this study, may be important in the future to help GPs care for their patients, by improving the process of stopping or reducing unnecessary medication.

Your answers will be kept anonymous. This means that neither your GP nor the study team can identify you or your answers.

You have the option of completing the questionnaire online or on paper. If you fill in the paper questionnaire, please return it to your GP in a sealed envelope. If you choose to complete the questionnaire online, please use this link or the QR-code above: ______

In this case, you do not need to return the hard copy of the questionnaire to your GP. We ask that you do not mention any names or addresses on the questionnaire to keep your anonymity.

This study is approved by the Ethics Committee of the Canton of Bern and complies with the legal requirements for research with anonymous medical data. The data will be securely stored electronically.

By answering "yes" to the question below and completing this questionnaire, you agree to participate in the study and the research team will collect your responses for the purpose of this study.

It will take about 15-30 minutes to complete the questionnaire.

Your help means a lot to us. Thank you again for taking the time to complete this questionnaire. If you have any questions or comments, please contact the study team:

Email: (Add email national coordinator)

Yours sincerely,

(Add name of national coordinator)

Prof. Sven Streit, PhD MD MSc

Principal Investigator of the LESS study

Institute of Primary Health Care (BIHAM)

University of Bern

© 2025 Vidonscky Lüthold R et al. *JAMA Network Open*.

Informed consent

Do you agree to participate in this study in which we will collect information about your medication use?

If you check "Yes", you agree to participate.

o Yes *(please continue to the next question)*

o No *(end of study participation)*

1) Questions about the inclusion criteria

1. How old are you (in years)?
   - o 65 years old or older *(please continue to the next question)*
   - o 64 years old or younger *(end of study participation, you are not eligible for this study*)

2. Do you regularly take 5 or more medications? (Regularly means: every day or most days for 30 days or more)
   - o Yes *(please continue to the next question)*
   - o No *(end of study participation, you are not eligible for this study*)

3. Do you live in (add country)
   - o Yes *(please continue to the next question)*
   - o No *(end of study participation, you are not eligible for this study*)

2) Socio-demographic questions

We will now ask you some questions to understand a bit more about you.

4. What is your gender?
   - o man
   - o woman
   - o other

5. What area do you live in?
   - o urban
   - o suburban
   - o rural

6. Do you live alone in your household?
   - o yes
   - o no

7. What is your living situation?
   - o Own your house or apartment
   - o Rented house or apartment

8. What is your highest completed education?
   - o None
   - o Primary school
   - o Secondary school (high school or vocational training)
   - o Third level education (university or equivalent training)

© 2025 Vidonscky Lüthold R et al. *JAMA Network Open*.

9. How do you make ends meet financially?
   - With great difficulty
   - With some difficulty
   - Quite easily
   - Without any problems

10. Where were you born?
    - In the country where I currently live
    - Other country: Please specify country___________________

11. What is your first language?
    - Official language of the country where I live in
    - Other language: Please specify language_________________

12. How confident are you filling out medical forms by yourself?
    - Not at all
    - A little bit
    - Somewhat
    - Quite a bit
    - Extremely

13. In general, how would you describe your health today?
    - Excellent
    - Very good
    - Good
    - Average
    - Poor

3) Questions about your GP

We will now ask you some questions about your GP.

14. Do you have your own GP/family doctor (definition: when you have a health problem, you usually consult the same family doctor, except in emergencies)?
    - Yes
    - No *(please go to Section 4) "questions about your use of medication")*
    - Unclear:
      - Reason: ____

15. How long have you been seeing this GP?
    - 0–9 years
    - 10–19 years
    - 20–29 years
    - 30+ years

16. My GP is:
    - man
    - woman
    - other

17. My GP's practice is:
    - In an urban area
    - In a suburban area
    - In the countryside

© 2025 Vidonscky Lüthold R et al. *JAMA Network Open*.

4) Questions about your medication use

Now we would like to learn more about your experiences with taking medications.

18. Do you prepare your medication by yourself?
    - Yes, I prepare and take it myself according to the prescription.
    - No, I receive support in preparing/taking my medication from relatives, home carers, or at the pharmacy for example.

19. Overall, I am satisfied with my current medications.
    - Strongly agree
    - Agree
    - Don't know
    - Disagree
    - Strongly disagree

20. How many different kinds of medications do you take regularly? (Regularly means daily or on most days of the week.) Please indicate the number of different kinds of medications.
    Number of different medications: ___________________________________

5) Questions about your attitude towards and decisions about medication

Now we are going to ask you questions on your thoughts about stopping or reducing the dose of medicines.

21. If my doctor said it was possible I would be willing to stop one or more of my regular medications.
    - Strongly agree
    - Agree
    - Don't know
    - Disagree
    - Strongly disagree

22. I would like to try stopping one of my medications to see how I feel without it.
    - Strongly agree
    - Agree
    - Don't know
    - Disagree
    - Strongly disagree

23. Thinking about your current medication list, are there any medications that you would like to stop taking or reduce the dose of?
    - Yes *(please continue to the next question)*
    - No, I am not considering stopping or reducing the dose of any medication. *(Please go to Question 25)*

© 2025 Vidonscky Lüthold R et al. *JAMA Network Open*.

24. In the following table, please state the name(s) of the medication(s) that you would consider stopping or reducing, and the reason why.
Any lines that are not applicable can be left empty.

**Name(s) of the medication(s) that you would consider stopping or reducing**	**Why did you choose this/these medication(s) to stop or reduce?**<br><br>*Please check all answers that apply*
**Name of the medication:**<br><br>____________________________	○ It causes side effects.<br>○ I do not benefit from it.<br>○ I do not like the medication.<br>○ The medication is too expensive.<br>○ It is inconvenient for me to take this medication.<br>○ The tasks involved in taking the medication(s) (e.g. blood glucose monitoring) are stressful for me.<br>○ I often forget to take this medication.<br>○ Other reason:____________________________
**Name of the medication:**<br><br>____________________________	○ It causes side effects.<br>○ I do not benefit from it.<br>○ I do not like the medication.<br>○ The medication is too expensive.<br>○ It is inconvenient for me to take this medication.<br>○ The tasks involved in taking the medication(s) (e.g. blood glucose monitoring) are stressful for me.<br>○ I often forget to take this medication.<br>○ Other reason:____________________________
**Name of the medication:**<br><br>____________________________	○ It causes side effects.<br>○ I do not benefit from it.<br>○ I do not like the medication.<br>○ The medication is too expensive.<br>○ It is inconvenient for me to take this medication.<br>○ The tasks involved in taking the medication(s) (e.g. blood glucose monitoring) are stressful for me.<br>○ I often forget to take this medication.<br>○ Other reason:____________________________
**Name of the medication:**<br><br>____________________________	○ It causes side effects.<br>○ I do not benefit from it.<br>○ I do not like the medication.<br>○ The medication is too expensive.<br>○ It is inconvenient for me to take this medication.<br>○ The tasks involved in taking the medication(s) (e.g. blood glucose monitoring) are stressful for me.<br>○ I often forget to take this medication.<br>○ Other reason:____________________________

After the table please continue to section 6 "additional questions about stopping medications and your willingness to do so".

© 2025 Vidonscky Lüthold R et al. *JAMA Network Open*.

25. You may not want to stop taking a medication or reduce the dose. Here are some reasons why. Which one(s) do you think are the most important reasons for not stopping a medication? (Please select all that apply)
    - The medicine is beneficial.
    - Taking the medicine for a long time so it is better not change it.
    - Taking several medications every day is manageable.
    - The medication does not cause side effects.
    - Medication(s) are not expensive.
    - Doctors only prescribe medication(s) that are necessary.
    - It is easier to take medications than to make healthy lifestyle changes.
    - Other reasons: ____

6) Stopping medications and your doctor's involvement:

We will now ask you some questions about how you would stop or reduce the dose of your medications with your doctor.

26. I feel comfortable talking to my doctor about changes to my medication
    - Strongly agree
    - Agree
    - Don't know
    - Disagree
    - Strongly disagree

27. Who would you talk to about stopping or reducing the dose of a medication*? (Please check all that apply)*
    - GP
    - Specialist
    - Pharmacist
    - Family and friends
    - Other

28. What would help you to stop or reduce the dose of a medication? *(Please check all that apply)*
    - A plan or instructions for stopping or reducing the dosage
    - The support of my GP
    - An alternative medication instead
    - An alternative such as a lifestyle change, physiotherapy
    - The option to restart the medicine if I feel I need to, or my symptoms return
    - Other:____

For each of the following, please select the statement that best aligns with your views.

29. What do you think about the medications you take?
    - My medications are important, they keep me alive and help me live well.
    - My medications do what they are supposed to do.
    - I don't really care much about my medications, I take them as my doctor tells me to.

30. How do you get information about your medications?
    - My doctor and I talk about my medications together.
    - I know about my medications – I ask my doctor or read the information leaflet or search online.
    - I don't know much about my medications.

© 2025 Vidonscky Lüthold R et al. *JAMA Network Open*.

31. How do you make decisions about your medications?
    - o I want to be informed, but I trust my doctor to make decisions about my medications.
    - o I make decisions about the medications I take, or share the decision with my doctor.
    - o Other people (e.g. my doctor or my partner) make decisions for me about my medications.

32. What do you think about the idea of stopping or reducing the dose of one or more of your medications?
    - o I would not like to stop any of my medications or reduce the dose.
    - o I wish I did not take so many medications and I would stop or reduce the dose of my medications if I could.
    - o If my doctor said that it is possible to stop or reduce the dose of a medication that would be ok with me.

7) Questions about your relationship to your family doctor

33. This section is about your relationship with your GP and your trust in them. Please indicate how strongly you agree with each of the statements. There are no right or wrong answers.

	Completely disagree	Disagree	Don’t know	Agree	Completely agree
Sometimes my GP cares more about what is convenient for them than about my medical needs.					
My GP is extremely thorough and careful.					
I completely trust my GP's decision about which medical treatments are best for me.					
My GP is completely honest about the different treatment options available for my health problem.					
All in all, I have complete trust in my GP.					

8) Final questions

34. Did anyone help you with completing this questionnaire?

- o Yes
    - o If yes: Who? *(please check the answer that applies)*
        - ▪ Relatives
        - ▪ Friends
        - ▪ GP
        - ▪ GP practice staff
        - ▪ Other: ____
    - o No

You had the opportunity to complete the questionnaire online or on paper. Please confirm that you **only completed one** of the versions of the questionnaire.

- o “I confirm that I only completed one of the versions of the questionnaire.”

Thank you for taking the time to complete the questionnaire.

[Please return this questionnaire to your GPs office as soon as possible.]

If you have any questions, please do not hesitate to contact us.

Yours sincerely,

(Add national coordinator)

Prof. Sven Streit and the LESS Study team

© 2025 Vidonscky Lüthold R et al. *JAMA Network Open*.
