# Supplementary material for: Older Adults’ Attitudes Toward Deprescribing in 14 Countries
Source: JAMA Netw Open. 2025 Feb 10;8(2):e2457498. doi: 10.1001/jamanetworkopen.2024.57498 (PMC11811803; doi:10.1001/jamanetworkopen.2024.57498)
Supplement: Supplement 2. — Data Sharing Statement [file jamanetwopen-e2457498-s002.pdf]

Data Sharing Statement

Vidonscky Lüthold. Older Adults' Attitudes Toward Deprescribing in 14 Countries. *JAMA Netw Open*. Published February 05, 2025. doi:10.1001/jamanetworkopen.2024.57498

Data
Data available: No

Additional Information
Explanation for why data not available: The data for this study are available to other researchers on request. The data will be made available for scientific research purposes, after the proposed analysis plan has been approved by the core study team. Data and documentation will be made available through a secure file exchange platform after approval of the proposal. In addition, a data transfer agreement must be signed (which defines obligations that the data requester must adhere to regarding privacy and data handling). For data access, please contact the corresponding author.
